# A Point Mutation in a Herpesvirus Co-Determines Neuropathogenicity and Viral Shedding

**DOI:** 10.3390/v9010006

**Published:** 2017-01-10

**Authors:** Mathias Franz, Laura B. Goodman, Gerlinde R. Van de Walle, Nikolaus Osterrieder, Alex D. Greenwood

**Affiliations:** 1 Department of Wildlife Diseases, Leibniz Institute for Zoo and Wildlife Research, Berlin 10315, Germany; 2 Department of Population Medicine and Diagnostic Sciences, Cornell University, Ithaca, NY 14850, USA; laura.goodman@cornell.edu; 3 Baker Institute for Animal Health, Cornell University, Ithaca, NY 14850, USA; grv23@cornell.edu; 4 Institut für Virologie, Freie Universität Berlin, Robert Von Ostertag-Str. 7 – 13, Berlin 14163, Germany; no.34@fu-berlin.de; 5 Department of Veterinary Medicine, Freie Universität Berlin, Oertzenweg 19b, Berlin 14163, Germany

**Keywords:** equine herpesvirus type 1, neuropathogenicity, viral shedding, trade-off hypothesis

## Abstract

A point mutation in the DNA polymerase gene in equine herpesvirus type 1 (EHV-1) is one determinant for the development of neurological disease in horses. Three recently conducted infection experiments using domestic horses and ponies failed to detect statistically significant differences in viral shedding between the neuropathogenic and non-neuropathogenic variants. These results were interpreted as suggesting the absence of a consistent selective advantage of the neuropathogenic variant and therefore appeared to be inconsistent with a systematic increase in the prevalence of neuropathogenic strains. To overcome potential problems of low statistical power related to small group sizes in these infection experiments, we integrated raw data from all three experiments into a single statistical analysis. The results of this combined analysis showed that infection with the neuropathogenic EHV-1 variant led to a statistically significant increase in viral shedding. This finding is consistent with the idea that neuropathogenic strains could have a selective advantage and are therefore systematically increasing in prevalence in domestic horse populations. However, further studies are required to determine whether a selective advantage indeed exists for neuropathogenic strains.

## 1. Introduction

Equine herpesvirus type 1 (EHV-1) is a ubiquitous alphaherpesvirus that can cause respiratory disease, abortion, neonatal foal death and equine herpes myeloencephalopathy (EHM), a neurological disease that can be lethal [1,2]. Although it is not well understood how EHM develops following EHV-1 infection, it has been shown that neuropathogenicity is significantly associated with a single nucleotide polymorphism in the viral DNA polymerase, resulting in a specific amino acid change; asparagine to aspartic acid (N/D_752_) [1,2,3]. To demonstrate the causality of this association, mutations were introduced in the backgrounds of representative D_752_ and N_752_ strains from Europe and North America, cloned as bacterial artificial chromosomes and then characterized thoroughly both in vitro and in vivo [4,5]. Although involvement of a variety of host, viral, and environmental risk factors are known or suspected, infections with D_752_ strains strongly increase the risk of developing EHM [1]. Based on two retrospective large-scale studies, it has been estimated that the odds of having EHM with a D_752_ strain were 490 times greater than those with a N_752_ strain [2].

Whether the neuropathogenic D_752_ strains are increasing in prevalence and what could enable such an increase are important and as yet unresolved questions. The D_752_ or the N_752_ at synonymous positions in DNA polymerases of other herpesviruses is highly conserved [3], although EHM has been considered rare in the past. Following worldwide EHM outbreaks in 2006–2007, the US Department of Agriculture classified EHM as a potentially emerging disease [6]. Related concerns that neuropathogenic D_752_ strains are spreading in domestic horse populations have been supported by a limited number of large-scale retrospective studies. These studies indicated that while N_752_ strains are more common [2,7], D_752_ strains have increased in prevalence in recent decades [8], which may suggest the existence of a selective advantage of neuropathogenic D_752_ strains. However, what could mediate such a selective advantage remains unclear. In contrast to the potential fitness benefits, D_752_ strains might suffer from fitness costs related to increased host death rates that result from neuropathogenicity [9].

D_752_ strains might have nevertheless a selective advantage if they have sufficiently increased transmission rates compared to N_752_ strains. EHV-1 transmission occurs via direct contact, fomites, or by inhalation of respiratory secretions [2]. Primary infections caused by EHV-1 occur at the respiratory epithelium and result in nasal shedding for 10 to 14 days. These infections typically result in life-long latent infections with subsequent viral reactivation and viral shedding during periods of stress [1]. Differences in transmission rates among strains can therefore have at least three causes: (1) the differences in the amount of nasal virus shedding during primary infections; (2) differences in the amount of nasal virus shedding at reactivation; and (3) differences in the rate of reactivation. Among these sources, previous studies have been limited to investigations of viral shedding during primary infections. Specifically, two experimental infection studies, which document that the D_752_ variant is more likely to cause neuropathogenicity, failed to find evidence that infections with the D_752_ variant cause statistically significant increased amounts of nasal virus shedding compared to the N_752_ variant [4,5]. These results were interpreted as suggesting the absence of a consistent selective advantage of the D_752_ variant and therefore were thought to indicate the absence of a systematic increase in the prevalence of neuropathogenic D_752_ strains.

Here, we re-examined data from the two previous studies to address the potential problem that the absence of statistically significant differences in shedding amounts reflected an actual absence of differences; and the absence of statistical significance could be related to low statistical power. To overcome potential problems of low statistical power related to small sample sizes, we integrated the original raw data from all three experimental infection studies into a single statistical analysis and re-assessed whether increased neuropathogenicity of the D_752_ variant coincides with increased viral shedding.

## 2. Materials and Methods

**Figure 1:**
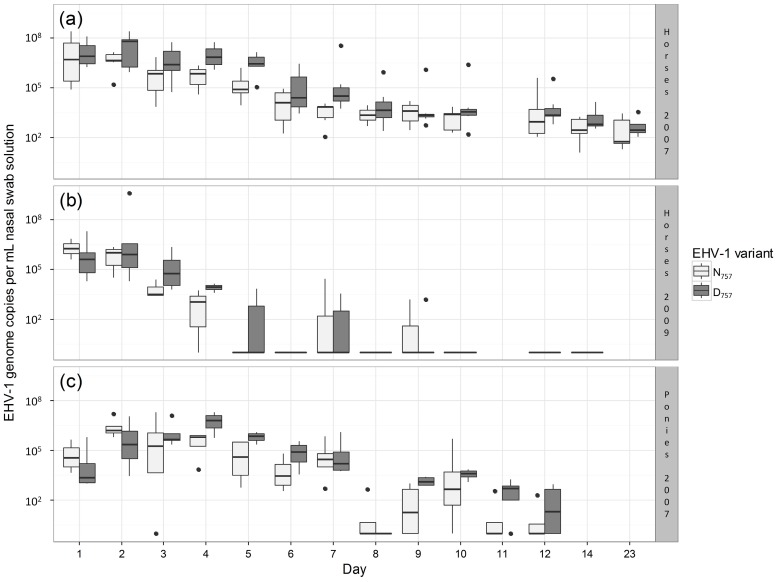
Boxplots illustrating the differences in amounts of nasal virus shedding between equine herpesvirus type 1 (EHV-1) variants (N/D_752_) across all days and animals for each experiment. (**a**) Experiment on 14 horses (seven infected with the N_752_ and seven infected with the D_752_ variant) conducted by Goodman et al. [4]; (**b**) Experiment on nine horses (three infected with the N_752_ and six infected with the D_752_ variant) conducted by Van de Walle et al. [5]; (**c**) Experiment on eight ponies (four infected with the N_752_ and four infected with the D_752_ variant) conducted by Goodman et al. [4]. If sampling took place on a given day, then all infected animals were sampled, with the exception of day 23 in the experiment on horses conducted by Goodman et al. where samples were restricted to six individuals infected with the N_752_ variant and four individuals infected with the D_752_ variant. In each boxplot, the bottom and top of the box indicate the first and third quartiles, and the band inside the box indicates the median. Upper (and lower) whiskers extend to the highest (and lowest) value that is within 1.5 times the inter-quartile range (i.e., the distance between the first and third quartiles). Data points beyond the end of the whiskers are plotted as dots [10]. Due to highly skewed distributions, the amount of nasal virus shedding was plotted on a logarithmic scale.

In our analysis, we integrated data on nasal virus shedding from infection experiments conducted by Goodman et al. [4] and Van de Walle et al. [5]. Both studies used a similar experimental design in which two groups of animals were infected with the N_752_ or D_752_ EHV-1 variant (Table 1). However, the performed experiments differed in specific details that include infection dose, breed of studied animals and number of infected animals. Goodman et al. conducted two separate experiments: (1) two groups of four Welsh Mountain ponies infected with 7 × 10^6^ plaque-forming units (PFUs) of the virus, and (2) two groups of seven mixed-breed horses infected with 1 × 10^7^ PFUs [4]. Van de Walle et al. conducted one experiment on a group of three and six horses (infected with the N_752_ and D_752_ variant, respectively), which were each inoculated with 1.5 × 10^7^ PFUs [5]. Following infection, nasal swabs were obtained from each animal at a daily or near-daily basis (Figure 1). For all three experiments, quantitative polymerase chain reaction was used to measure the amount of nasal virus shedding and estimate the viral genome copy number in nasal swabs. All three experiments were performed in accordance with relevant guidelines and regulations. The experimental protocols on horses were approved by the Cornell University Institutional Animal Care and Use Committee [4,5]. The experimental protocol on ponies was approved by the UK Home Office [4].

In our statistical analysis, we aimed to test whether amounts of nasal virus shedding differed between the two EHV-1 variants. We focused only on shedding amount and did not analyze shedding duration because the majority of animals (17 out of 31) were still shedding on the last respective sampling day. To maximize statistical power in our analysis of nasal virus shedding, we pooled all available raw data from the three experiments. Accordingly, our data set consisted of 382 data points, where each data point was related to one individual measure of nasal virus shedding (Table 1, Figure 1). This approach required the analysis to appropriately control for non-independence of data points and related variation in shedding amounts that emerged from (1) consistent differences among animals, which might, for example, relate to physiological or genetic differences; (2) systematic differences among the three experiments, which could, for example, relate to differences in infection treatment or horse breeds; and (3) temporal variation in shedding over the course of each experiment. To address these issues, we used a linear mixed model, in which each nasal swab was treated as a single data point with the amount of nasal virus shedding as the response variable. The EHV-1 variant (N_752_ vs. D_752_) was treated as a fixed effect, which tested whether shedding differed between the two EHV-1 variants. To account for variation in nasal virus shedding among experiments and over time within each experiment, we included a categorical predictor ‘experiment_day’ as a fixed effect in our model. This predictor combines information on the experiment and day after infection, which resulted in 37 unique levels (13 + 12 + 12 days). To account for potential differences among animals and non-independence of data points, the identity of each animal was included as a random effect. To prevent violations of model assumptions, we log-transformed the response variable. After this transformation, visual inspections of the model output did not indicate any violations of assumptions regarding the normality and homogeneity of error variances. Model fitting and analysis were performed using the package ‘glmmADMB’ [11] in the statistical software R [12]. Statistical significance was assessed based on a two-sided test with an α-level of 0.05. The datasets supporting this article are available from the Dryad Digital Repository [13].

## 3. Results

Results of our combined analysis showed that the amount of EHV-1 nasal shedding was significantly increased in animals infected with the D_752_ variant compared with infection with the N_752_ variant (*p* = 0.001). The model indicated that infections with the D_752_ variant led, on average, to a four-fold higher amount of nasal EHV-1 shedding compared to infections with the N_752_ variant (Figure 1). To control for potentially confounding effects of combining data on mixed-breed horses and Welsh Mountain ponies, we also performed an analysis that excluded the pony data (Figure 1c). The analysis of this reduced data set did not qualitatively change our results: the differences between both variants remained significant (*p* = 0.004) with an estimated 3.9-fold increase in the amount of nasal virus shedding by animals infected with the neuropathogenic D_752_ variant.

## 4. Discussion

In contrast to previously conducted and separately analyzed studies, we found support for the hypothesis that, compared to the non-neuropathogenic N_752_ variant, primary infection with neuropathogenic D_752_ results in increased nasal virus shedding. This finding indicates that the absence of statistical differences in shedding amounts in previous studies was related to low statistical power. Given the constraints of performing infection experiments on a large number of animals, our findings emphasize (1) the importance of using advanced statistical modeling approaches and (2) the usefulness of pooling raw data from individual experiments.

Our finding of increased nasal virus shedding of the D_752_ variant following experimental EHV-1 infections of equids is consistent with the idea that D_752_ strains could have a selective advantage and are therefore systematically increasing in prevalence in domestic horse populations [8]. However, our finding is not sufficient to determine whether a fitness advantage indeed exists for D_752_ variants, which would require more precise information on infection-induced host death rates and rates of viral reactivation during latent infections. In addition, due to effects on host death and transmission rates, the fitness of different EHV-1 strains might be strongly determined by within-host interactions. Co-infection with both variants is possible [1,7], which suggests that within-host interactions between different strains might occur, which could impact strain fitness and related evolutionary dynamics [14,15]. We are not aware of any study that has investigated within-host interactions between the two variants. Nevertheless, there is some indication that the neuropathogenic variant has a competitive advantage regarding within-host interactions. Compared to the N_752_ variant, infections with the D_752_ variant led to higher levels of viremia and virus-neutralizing antibody titers [4,5]. This finding is consistent with D_752_ eliciting a higher immune response and being more resistant to the response than the N_752_ variant [16,17].

A growing body of research on this polymorphism has attempted to elucidate the biological mechanisms connecting DNA polymerase activity to EHM. There is a general consensus that magnitude and duration of viremia are central aspects of the disease ([18]; reviewed by [19]), with secondary fevers commonly seen in neurologic cases. Replication kinetics in cultured fibroblasts and epithelial cells were similar between variants [4]. Studies in primary respiratory epithelial and brain endothelial model systems have elucidated the ability of D_752_ strains to modulate immune evasion [20,21]. In nasal mucosal explants, D_752_ was more effective at crossing epithelial boundaries [22], but less effective in vaginal mucosal explants [23]. The ability to infect migratory leukocytes may be a key aspect of EHM progression, with high levels of nasal virus shedding one of the consequences.

Our finding that neuropathogenicity is linked to nasal virus shedding is consistent with assumptions underlying the trade-off hypothesis—the currently dominant but still controversial hypothesis of virulence evolution [24,25,26]. This hypothesis assumes that virulence evolution is crucially influenced by a mechanistic link between virulence (defined as increased host death rate) and pathogen transmission: decreasing virulence constrains pathogen shedding and the related rate at which pathogens are transmitted to susceptible hosts. This interdependence generates an evolutionary trade-off for pathogens because the ideal state of low virulence and high transmission rate becomes impossible to reach. Instead, fitness benefits of reducing virulence come at a fitness cost of a reduced transmission rate. Although theoretically appealing, there is currently only limited empirical support for the underlying assumption of virulence–transmission trade-offs [24,25,26,27,28,29,30,31,32,33,34].

In the absence of reliable quantifications of virulence and transmission rates of different EHV-1 strains, it is not possible to directly assess the validity of the trade-off hypothesis for EHV-1. Nevertheless, our finding of increased and nasal virus shedding for the neuropathogenic EHV-1 variant is fully consistent with this hypothesis. Conceptual descriptions and formal implementations of the trade-off hypothesis usually assume that pathogens can evolve different levels of virulence (i.e., increased host death rate) and transmission along a continuous scale [24,25,26]. In contrast, the currently existing variation in EHV-1 neuropathogenicity seems to be mainly caused by a single nucleotide polymorphism, which suggests that a continuous variation in virulence and transmission might be impossible in this system. As a consequence, it could be possible that there is no single optimal virulence in this system and that instead both variants permanently coexist [5].

## Figures and Tables

**Table 1:** Overview of analyzed data.

Experiment	Number of Data Points	Number of Sampling Days	Number of Animals Infected		Estimated Viral Genome Copy Numbers ^1^	
			N_752_	D_752_	N_752_	D_752_
Horses 2007 [4]	178	13	7	7	5.2 × 10^6^ ± 3.1 × 10^7^	1.2 × 10^7^ ± 3.6 × 10^7^
Horses 2009 [5]	108	12	6	3	3.5 × 10^5^ ± 1.2 × 10^6^	5.2 × 10^7^ ± 4.4 × 10^8^
Ponies 2007 [4]	96	12	4	4	9.5 × 10^5^ ± 3.7 × 10^6^	1.4 × 10^6^ ± 4.0 × 10^6^

^1^ Mean ± standard deviation (SD).
